# Diabetic kidney disease versus non‐diabetic kidney disease in type 2 diabetic patients on dialysis: An observational cohort

**DOI:** 10.1002/edm2.281

**Published:** 2022-04-30

**Authors:** Arnaud Delautre, Thierry Hannedouche, Cécile Couchoud, José Guiserix, Damiano Cerasuolo, François Chantrel, Jonas Martzloff, Nicolas Keller, Thierry Krummel, the REIN registry

**Affiliations:** ^1^ Service de Néphrologie et Dialyse Hôpitaux Universitaires de Strasbourg Strasbourg France; ^2^ Faculté de Médecine Université de Strasbourg Strasbourg France; ^3^ Agence de Biomedecine Paris France; ^4^ Centre Hospitalier Universitaire de La Réunion Mayotte France; ^5^ Centre Hospitalier de Caen Caen France; ^6^ Service de Nephrologie Centre Hospitalier Emile Muller de Mulhouse Mulhouse France

**Keywords:** chronic kidney disease, diabetic kidney disease, mortality, transplantation, type 2 diabetes

## Abstract

**Background:**

All chronic kidney diseases in diabetic patients are not diabetic kidney diseases. The objective was to compare the clinical characteristics, survival and access to transplantation in diabetic patients starting dialysis and classified either as diabetic kidney disease (DKD) or non‐diabetic kidney disease in diabetic patients (NDKD).

**Methods:**

We used the nationwide French REIN registry to analyse baseline clinical characteristics at dialysis inception and outcomes defined as kidney transplantation, deaths and their causes. The probability of death or transplantation was analysed using a multivariate Cox model and the Fine and Gray competing for risk model (sdHT).

**Results:**

We included 65,136 patients from January 2009 to December 2015 with a median follow‐up of 31 months. The cumulative incidence of kidney transplantation over eight years was 46.9% (44.8–48.9) in non‐diabetic patients (ND), higher than the 19.3% (17.5–21.2) in the DKD group and 22.2% (18.4–26.7) in the NDKD group. The risk of death was significantly higher in the NDKD group than in the DKD group, even after accounting for the competing risk of transplantation (NDKD/sdHR 1.22; 95%CI 1.18–1.27; *p* < 0.005 vs. DKD/sdHR 1.12; 95%CI 1.08–1.16; *p *< 0.005 with adjustment for age, sex, major adverse cardiovascular events, cancer and chronic respiratory failure, compared to ND).

**Conclusions:**

In diabetic patients starting dialysis, patients in the DKD group had reduced access to kidney transplantation. NDKD patients had a higher risk of mortality than DKD. The distinction between DKD and NDKD should be accounted for in the plan of care of diabetic patients starting dialysis.

## Graphical abstract

What is already known about this subject? Diabetes is the leading cause of end‐stage kidney disease throughout the world. All chronic kidney diseases in diabetics are not diabetic kidney diseases (about 25%). There are few and conflicting data on the prognosis significance of this distinction. What this study adds: We found marked clinical differences between DKD and NDKD groups: more vascular‐related complications, more insulin treatment and more disability in DKD, more cancer, liver diseases and heart rhythm disorders in NDKD patients. Survival was better overall in patients in the DKD versus the NDKD group. Some patients in the NDKD group had more access to kidney transplantation, suggesting heterogeneity in this group. Some clinical factors were found to be associated with reduced access to kidney transplantation, including insulin treatment and congestive heart failure. What impact this may have on practice or policy?. The different outcomes should be accounted for in the plan of care of diabetic patients starting dialysis, especially when planning transplantation, and question a role for expanding indications of kidney biopsies to classify subgroups of diabetic people more accurately.

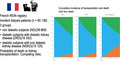

**Abbreviations**
BMI: body mass index
csHR: Cause‐specific hazard ratio
DKD: Diabetic kidney disease
ESA: Erythropoiesis‐stimulating agent
ESKD: End‐stage kidney disease
HR: Hazard ratio
KRT: Kidney replacement therapy
MACE: Major adverse cardiovascular events
ND: Non‐diabetic patients
NDKD: Non‐diabetic kidney disease
sdHR: Subdistribution hazard ratio

## 1 INTRODUCTION

Worldwide, the number of subjects with diabetes has doubled in the last 20 years, reaching 415 million adults in 2015.^1^ Diabetes, primarily type 2 diabetes, is currently the leading cause of end‐stage kidney disease (ESKD) worldwide, with significant variability across countries.^2^ However, in patients with diabetes and chronic kidney disease, the underlying cause of kidney damage is rarely known with confidence. Ageing and comorbidities, particularly hypertension, obesity and congestive heart failure, also contribute to the risk of chronic kidney disease,^3^ and the coexistence of two types of nephropathy is not uncommon. Besides, the clinical manifestations of kidney disease in people with diabetes have changed over time, with an increasing prevalence of reduced estimated glomerular filtration (eGFR) rate with low‐grade albuminuria.^4^ In the National Health and Nutrition Examination Survey (NHANES), only 24% of the total prevalence of chronic kidney disease in people with diabetes was attributable to diabetes after considering age, gender and ethnicity.^5^

In France, diabetes was diagnosed in 40% of incident dialysis patients but stated as the cause of ESKD in 21.4% (54% of type 2 diabetic patients).^6^, ^7^ These paradoxical findings suggest two distinct entities, namely diabetes as comorbidity superimposed on another nephropathy (non‐diabetic kidney disease in diabetic patient, NDKD) and true DKD related to long‐standing diabetes and poor metabolic control.

Survival among dialysis patients with diabetes is lower than the survival of non‐diabetic dialysis patients, in part due to a higher number of comorbidities.^6^, ^8^ It was suggested that patients with DKD could have more extensive vascular disease in the kidneys and elsewhere than NDKD patients, which would affect their prognosis. However, only scarce data are available and reported outcomes have been conflicting.^9^, ^10^

The objective of our study was to analyse the clinical characteristics, survival and access to transplantation in diabetic patients starting dialysis and classified either as DKD or NDKD by their nephrologist and by comparison with non‐diabetic patients.

## 2 PATIENTS AND METHODS

This observational cohort study relied on the nationwide REIN registry, which includes all ESKD patients on kidney replacement therapy (KRT)—either dialysis or transplantation—living in France.^7^, ^11^ The details of its organizational principles and quality control have been described elsewhere.^7^ The study was nested in the REIN registry approved by the CCTIRS, the CNIL and the Scientific Council of the Agence de Biomedecine. All patients gave their informed consent.

We included all adults (> 20 years) starting KRT from January 2009 to December 2015, except for type 1 diabetic patients and pre‐emptive transplants. The following clinical data were extracted from the registry at initiation of dialysis: age, sex, body mass index (BMI), serum albumin, haemoglobin, estimated GFR at dialysis inception, diabetes status, cause of kidney disease coded as a primary or secondary cause, kidney biopsy, major adverse cardiovascular events (MACE) (defined as at least one among the following cardiovascular comorbidities: coronary artery disease, congestive heart failure, arrhythmia, abdominal aortic aneurysm, peripheral arterial disease, stroke), disabilities (amputation, hemiplegia or paraplegia, severe impairment of vision, behavioural disorder), smoking status chronic respiratory failure, cancer, liver disease, other transplants than kidneys (heart‐lung, heart, lung, liver, bone or stem cell), HIV status, setting of the first dialysis (emergency, on catheter, or in the ICU), dialysis modality (haemodialysis or peritoneal dialysis), previous treatments (erythropoiesis‐stimulating agent (ESA), insulin), time between fistula placement and initiation of haemodialysis and number of consultation by a nephrologist before inception of dialysis.

From 2009 to the end of 2016, the following outcomes were collected**:** kidney transplantation, waiting time before kidney transplantation, death and causes of death. The follow‐up was a maximum of 8 years and at least one year for the last patient included.

DKD was differentiated from NDKD using the primary diagnoses and secondary diagnoses of nephropathy. If one of these two diagnoses was classified as DKD, then the diagnosis of DKD was retained, as opposed to NDKD. Non‐diabetic patients were classified as ‘ND’.

### 2.1 Statistics

First, we compared the three discrete groups: non‐diabetic (ND), diabetic kidney disease (DKD) and non‐diabetic kidney disease in diabetic patients (NDKD). Characteristics at the initiation of dialysis and during the follow‐up were compared with the Student's test or the Chi2 test as appropriate. Logistic regression was performed to adjust for age and sex. The difference was considered significant for a *p*‐value < 0.05.

Outcomes were studied by plotting the Kaplan‐Meier survival curves of the three groups. A log rank test was performed to compare curves over an eight‐year follow‐up. The cumulative incidence of specific causes of death was analysed with a subdistribution hazard (sdHT) Fine and Gray's model to account for the competing risks between various causes of death. The effect of CRD on the specific causes of death was analysed with an adjusted (cause‐specific) Cox proportional hazard regression (csHR) censored at other cause of death,^12^ adjusted for the variables of confusion (age, sex, MACE, cancer, chronic respiratory failure) which were independent predictors of mortality in the REIN cohort.^7^

The relationship between the characteristics at the initiation of dialysis and the probability of transplantation in the two DKD and NDKD groups was analysed by a multivariate Cox model (backward selection of determinants with a *p* < 0.20 in the univariate analysis).

Statistics were performed with STATA v14 software (Statacorp LLC).

## 3 RESULTS

Of a cohort of 70,540 patients starting KRT between 2009 and 2015, we excluded 851 patients under 20 years old, 1,629 patients with type 1 diabetes and 2,318 pre‐emptive transplants (flowchart Figure S1). The remaining 65,136 patients included 38,669 in the ‘non‐diabetic’ (ND) group, 16,342 in the ‘DKD’ group and 10,125 in the ‘NDKD’ group, or 59.4%, 24.1% and 15.5% of the cohort, respectively.

The mean age was 67 years in the ND group, 71 in the DKD group and 73 in the NDKD group (*p* < 0.05 after adjustment for gender) (Table 1). The history of MACE was 48% in the ND group versus 72% in the DKD group and 70% in the NDKD group (*p* < 0.05). There was a slight albeit significant difference between DKD and NDKD for coronary artery disease (37% versus 35%), arrhythmias (25% versus 30%), peripheral arterial disease (34% versus 28%), abdominal aortic aneurysms (2% versus 5%, *p* < 0.05), but not for congestive heart failure and stroke. There was 7% of cancer in the DKD group versus 12% in the NDKD group (*p *< 0.05). There were more liver diseases and more ‘other transplants than kidney’ in the NDKD group compared to the DKD group 6% versus 4.5%, (*p *< 0.05) and 2.1% vs. 0.5%, (*p *< 0.05), respectively.

**TABLE 1:** Patient characteristics at the start of kidney replacement therapy according to diabetic kidney disease (DKD) or non‐diabetic kidney disease (NDKD) coding

Characteristics	Non‐ diabetic	DKD	NDKD	*p*		
	*n* = 38,669 59.4%	*n* = 16,342 25.1%	*n* = 10,125 15.5%	*p* a	*p* b	*p* c
Age, mean ± SD	67 ± 17.0	71 ± 10.5	73 ± 10.9	<0.005	<0.005	<0.005
Men, %	63%	61%	66%	<0.005	<0.005	<0.005
BMI, mean ± SD	25 ± 5.2	29 ± 6.3	28 ± 8.3	<0.005	<0.005	<0.005
Serum Albumin, mean ± SD	33.6 ± 6.6	32.8 ± 6.0	32.7 ± 6.5	<0.005	<0.005	NS
Haemoglobin, mean ± SD	10.1 ± 1.8	10.1 ± 1.6	10.1 ± 1.6	<0.005	<0.05	NS
eGFR, mean ± SD	9.8 ± 8.1	10.5 ± 6.7	11.4 ± 9.8	<0.005	<0.005	<0.005
Renal biopsy, %	21.5%	8%	15.5%	<0.005	<0.005	<0.005
MACE	48%	72%	70%	<0.005	<0.005	<0.005
MACE, mean ± SD	0.89 ± 1.2	1.44 ± 1.3	1.48 ± 1.3	<0.005	<0.005	NS
Ischemic heart disease, %	19%	37%	35%	<0.005	<0.005	<0.005
Congestive heart failure, %	22%	33%	35%	<0.005	<0.005	NS
Arrhythmia, %	21%	25%	30%	<0.005	<0.005	<0.005
Abdominal aortic aneurysm, %	5%	2%	5%	<0.005	<0.005	<0.005
Peripheral arterial disease, %	13%	34%	28%	<0.005	<0.005	<0.005
Stroke, %	9%	14%	14%	<0.005	<0.005	NS
Disabilities						
Disability, %	10%	20%	16%	<0.005	<0.005	<0.005
Amputation, %	0.5%	4.5%	2.5%	<0.005	<0.005	<0.005
Hemiplegia or paraplegia, %	1%	2%	2%	<0.005	<0.005	NS
Severe vision impairment, %	1%	7%	3%	<0.005	<0.005	<0.005
Severe behavioural disorders, %	3%	3%	4%	NS	<0.005	<0.05
Other comorbidity						
Smoking, %	39%	41%	46%	<0.005	<0.005	<0.005
Chronic respiratory disease, %	11%	15%	19%	<0.005	<0.005	<0.005
Cancer, %	14%	7%	12%	<0.005	<0.005	<0.005
Liver disease, %	4%	4.5%	6%	<0.005	<0.005	<0.005
Other transplantation than kidney, %	1.5%	0.5%	2.1%	<0.005	<0.005	<0.005
HIV infection or AIDS	1%	0.4%	0.6%	<0.005	NS	<0.005
Treatment						
First dialysis in emergency, %	29%	31%	33%	<0.005	<0.005	<0.05
First HD on catheter, %	53%	53%	59%	NS	<0.005	<0.005
First HD in intensive care unit, %	9%	9%	11%	NS	<0.005	<0.005
First KRT modality: HD Vs PD, %	88%	91%	90%	<0.005	<0.005	<0.05
ESA treatment, %	46%	53%	48%	<0.005	NS	<0.005
Insulin treatment, %		79%	56%			<0.005
Time between fistula and HD (month), mean ± SD	6.3 ± 15.4	5.9 ± 11.9	5.9 ± 12.5	<0.005	<0.05	NS
Number of visit by a nephrologist, mean ± SD	3.9 ± 3.4	4.2 ± 3.1	3.8 ± 3.2	<0.005	NS	<0.005

Abbreviations: AIDS, acquired immunodeficiency syndrome; DKD, diabetic kidney disease; HD and PD, haemodialysis and peritoneal dialysis; HIV, human immunodeficiency virus; KRT, kidney replacement therapy; MACE, major adverse cardiovascular events; NDKD, non‐diabetic kidney disease; NS, no significance difference; Smoking, current smoker and ex‐smoker.

^a^ *p*: difference between ‘non‐diabetic’ and DKD, adjusted for gender and age.

^b^ *p*: difference between ‘non‐diabetic’ and NDKD, adjusted for gender and age.

^c^ *p*: difference between ‘DKD’ & NDKD, adjusted for gender and age.

**FIGURE 1:**
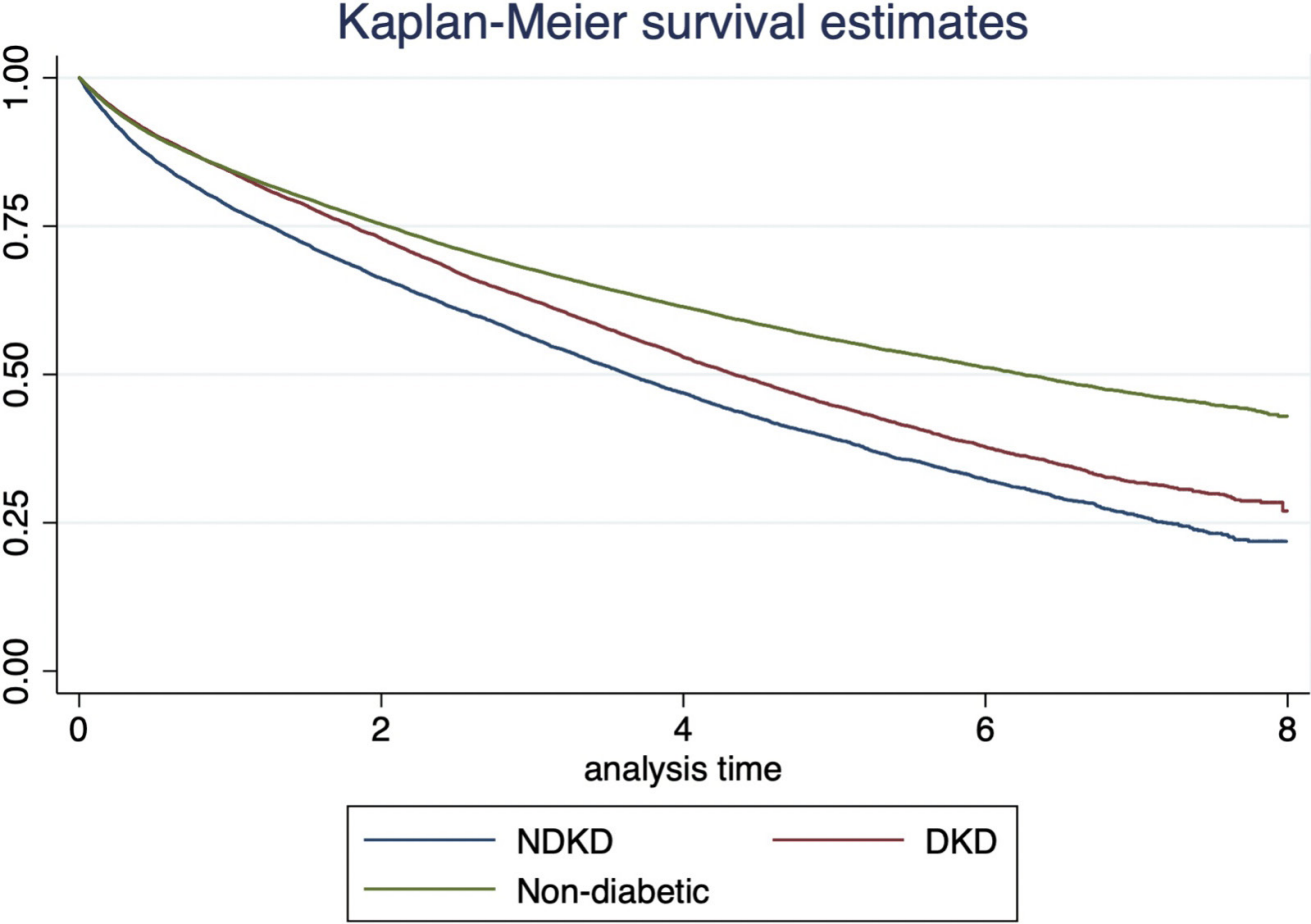
Survival analysis. Comparison of diabetic kidney disease (DKD), non‐diabetic kidney disease (NDKD) and non‐diabetic groups [Colour figure can be viewed at wileyonlinelibrary.com]]

A total of 28,149 patients died (43%). In the NDKD group, the 2‐year death rate was 33.9% (32.9–34.8) versus 27.1% (26.4–27.8) in the DKD group. At six years, there were 62% of deaths in the DKD group vs. 68% in the NDKD group. In the Kaplan‐Meier survival curve (Figure 1), the difference between the DKD vs. NDKD curves was significant (*p *< 0.05).

The risks of death in the different groups were compared by a multivariate Cox model (Table S1). After adjusting for age, gender, MACE, cancer and chronic respiratory failure, there was a 10% increased risk of death in the NDKD vs. DKD group compared to non‐diabetic patients (HR 1.21; 95%CI 1.17–1.26; *p *< 0.05 vs. HR 1.11; 95%CI 1.07–1.15; *p *< 0.05, respectively). The leading causes of death in DKD and NDKD groups are summarized in Table S2.

We studied the association between the clinical characteristics at dialysis inception and death in the NDKD vs the DKD group (Table 2). The determinants the most strongly associated with the risk of death were: liver disease (HR 1.60; 95%CI 1.29–1.98; *p *< 0.005), cancer (HR 1.57; 95%CI 1.36–1.80; *p *< 0.005), arrhythmias (HR 1.34; 95%CI 1.21–1.48; *p* < 0.005) and disabilities (HR 1.31; 95%CI 1.11–1.55; *p* < 0.005).

**TABLE 2:** Clinical determinants associated with the probability of death in diabetic patients starting dialysis. Multivariate analysis with the Cox model and with a competing risk model; calculation of sdHR (subdistribution hazard ratio) and csHR (cause‐specific hazard ratio)

Characteristics	HR	CI	*p*	csHR	CI	*p*	sdHR	CI	*p*
Age	1.04	1.03–1.04	<0.001	1.04	1.03–1.04	<0.001	1.04	1.04–1.05	<0.001
Female gender	0.92	0.80–1.06	0.251	0.92	0.80–1.06	0.262	0.93	0.80–1.07	0.289
BMI (body mass index) (per 1 unit)	1.02	1.01–1.03	<0.001	1.02	1.01–1.03	<0.001	1.02	1.02–1.03	<0.001
Serum albumin (per g/L)	0.98	0.97–0.99	<0.001	0.98	0.97–0.99	<0.001	0.98	0.97–0.99	<0.001
Haemoglobin (per g/dL)	0.99	0.96–1.02	0.430	0.99	0.96–1.02	0.408	0.99	0.96–1.02	0.420
eGFR	1.01	1.00–1.01	0.011	1.01	1.00–1.01	0.024	1.01	1.00–1.01	0.027
Renal biopsy	0.89	0.76–1.03	0.107	0.89	0.77–1.03	0.124	0.89	0.76–1.04	0.136
MACE	1.08	0.93–1.26	0.299	1.07	0.92–1.25	0.397	1.09	0.93–1.27	0.283
Ischemic heart disease	1.08	0.98–1.20	0.137	1.09	0.98–1.20	0.117	1.09	0.98–1.21	0.111
Congestive heart failure	1.27	1.15–1.41	<0.001	1.28	1.15–1.42	<0.001	1.28	1.15–1.42	<0.001
Arrhythmia	1.34	1.21–1.48	<0.001	1.32	1.19–1.47	<0.001	1.34	1.20–1.49	<0.001
Abdominal aortic aneurysm	1.07	0.85–1.35	0.566	1.07	0.85–1.35	0.543	1.08	0.86–1.34	0.522
Peripheral arterial disease	1.27	1.15–1.41	<0.001	1.27	1.14–1.41	<0.001	1.30	1.17–1.45	<0.001
Stroke	1.02	0.90–1.16	0.769	1.02	0.90–1.16	0.756	1.03	0.90–1.18	0.626
Disabilities									
Disability	1.31	1.11–1.55	0.002	1.30	1.10–1.54	0.003	1.32	1.09–1.60	0.005
Amputation	1.22	0.95–1.58	0.124	1.20	0.93–1.55	0.164	1.23	0.92–1.66	0.166
Hemiplegia or paraplegia	0.88	0.58–1.27	0.445	0.88	0.59–1.32	0.542	0.88	0.55–1.42	0.610
Severe vision impairment	0.76	0.61–0.96	0.022	0.76	0.60–0.96	0.022	0.75	0.59–0.96	0.021
Severe behavioural disorders	0.99	0.75–0.32	0.963	0.97	0.73–1.29	0.854	1.00	0.73–1.38	0.983
Other comorbidity									
Smoking	1.13	1.02–1.26	0.023	1.11	0.99–1.24	0.066	1.12	0.99–1.25	0.054
Chronic respiratory Disease	1.27	1.14–1.43	<0.001	1.26	1.12–1.42	<0.001	1.25	1.11–1.41	<0.001
Cancer	1.57	1.36–1.80	<0.001	1.53	1.33–1.76	<0.001	1.58	1.35–1.85	<0.001
Liver disease	1.60	1.29–1.98	<0.001	1.59	1.28–1.98	<0.001	1.64	1.31–2.04	<0.001
Other transplantation than kidney	1.35	0.93–1.95	0.113	1.63	1.12–1.36	0.010	1.50	1.05–2.14	0.027
HIV infection or AIDS	0.38	0.09–1.52	0.173	0.41	0.10–1.63	0.203	0.34	0.79–1.46	0.147
Treatment									
First dialysis in emergency	1.02	0.91–1.15	0.695	1.04	0.92–1.17	0.510	1.05	0.92–1.19	0.492
First HD on catheter	1.07	0.96–1.19	0.227	1.07	0.96–1.19	0.232	1.08	0.97–1.21	0.160
First HD in intensive care unit	0.99	0.82–1.19	0.924	0.97	0.81–1.18	0.787	0.98	0.81–1.21	0.873
First KRT modality: HD Vs PD	1.15	0.63–2.12	0.643	1.27	0.67–2.39	0.467	1.28	0.67–2.43	0.452
ESA treatment	1.04	0.95–1.14	0.407	1.05	0.95–1.15	0.349	1.04	0.95–1.15	0.391
Insulin treatment	1.08	0.97–1.19	1.161	1.06	0.96–1.18	0.258	1.08	0.97–1.20	0.172
Time between fistula and HD (month)	1.00	1.00–1.00	0.827	1.00	0.99–1.00	0.568	1.00	0.99–1.00	0.721

Abbreviations: AIDS, acquired immunodeficiency syndrome; DKD, diabetic kidney disease; ESA, erythropoietin‐stimulating agent; HD and PD, haemodialysis and peritoneal dialysis; HIV, human immunodeficiency virus; KRT, kidney replacement therapy; MACE, major adverse cardiovascular events; NDKD, non‐diabetic kidney disease; smoking, current smoker and ex‐smoker.

The cumulative incidence of kidney transplantation over eight years was 46.9% (44.8–48.9) in ND patients, much higher than the 19.3% (17.5–21.2) in the DKD group and 22.2% (18.4–26.7) in the NDKD group. The mean waiting time for renal transplantation was 3.0 ± 1.7 years in the DKD group and 2.7 ± 1.6 years in the NDKD group. The mean age at the time of transplantation was 63 ± 8.5 years in the DKD group, 62 ± 10.4 in the NDKD group and 52 ± 14 in non‐diabetic patients (Table 3).

**TABLE 3:** Outcomes of patients coded as non‐diabetics, diabetic kidney disease (DKD) and non‐diabetics kidney disease (NDKD): probability of transplantation and death

Pronostic	Non‐diabetic	DKD	NDKD
	*n* = 38 669	*n* = 16 342	*n* = 10 125
Death			
Died after 1 year of follow‐up, % (CI)	15.6% (15.2–16.0)	15.8% (15.2–16.4)^a^	21.8% (21.0–22.6)^b^, ^c^
Died after 2 year of follow‐up, % (CI)	24.7% (24.3–25.2)	27.1% (26.4–27.8)^a^	33.9% (32.9–34.8)^b^, ^c^
Died after 4 year of follow‐up, % (CI)	38.6% (38.1–39.2)	47.1% (46.2–48.0)^a^	53.1% (52.0–54.3)^b^, ^c^
Died after 6 year of follow‐up, % (CI)	48.9% (48.2–49.5)	62.3% (61.2–63.3)^a^	67.8% (66.4–69.1)^b^, ^c^
Died after 8 year of follow‐up, % (CI)	57.0% (55.8–58.2)	73.0% (69.9–76.1)^a^	78.1% (76.1–80.1^b^, ^c^
Kidney transplantation			
Transplantation after 1 year of follow‐up, % (CI)	6.4% (6.1–6.7)	1.2% (1.0–1.3)^a^	1.7% (1.5–2.0)^b^, ^c^
Transplantation after 2 years of follow‐up, % (CI)	15.7% (15.3–16.1)	4.0% (3.7–4.4)^a^	4.9% (4.4–5.5)^b^, ^c^
Transplantation after 4 years of follow‐up, % (CI)	31.1% (30.4–31.7)	10.6% (9.9–1.1)^a^	12.3% (11.4–13.3)^b^, ^c^
Transplantation after 6 years of follow‐up, % (CI)	39.9% (39.1–40.8)	15.7% (14.6–16.8)^a^	17.2% (15.7–18.8)^b^, ^c^
Transplantation after 8 years of follow‐up, % (CI)	46.9% (44.8–48.9)	19.3% (17.5–21.2)^a^	22.2% (18.4–26.7)^b^, ^c^
Kidney transplant listing, %	33.5%	15.4%^a^	14.7%^b^, ^c^
Duration before kidney transplantation (years), mean ± CI	2.5 ± 1.6	3.0 ± 1.7^a^	2.7 ± 1.6^b^, ^c^
Age at time of transplantation (years), mean ± CI	52 ± 14.0	63 ± 8.5^a^	62 ± 10.4^b^, ^c^
Time before registration (years), mean ± CI	0.5 ± 1.4	1.0 ± 1.3^a^	0.8 ± 1.4^b^, ^c^

Note

NS: no significant difference, CI: 95% confidence interval.

Abbreviations: DKD, diabetic kidney disease; NDKD, non‐diabetic kidney disease.

^a^ *p* < 0.005 between ‘non‐diabetic’ and DKD, adjusted for age and gender.

^b^ *p* < 0.005 between ‘Non‐diabetic’ and NDKD, adjusted for age and gender.

^c^ *p* < 0.005 between DKD and NDKD, adjusted for age and gender.

As the access to transplantation was marginally better in the NDKD than in the DKD group, we further studied the clinical determinants of renal transplantation in the two groups (Table S4). Higher serum albumin at inception and organ transplantation other than the kidney were associated with a higher probability of kidney transplantation in both groups. Conversely, high BMI, first dialysis on a catheter, advanced age, peripheral arterial disease, cancer and disability were associated with a lower probability of transplantation in both DKD and NDKD. Congestive heart failure decreased the probability of transplantation in the NDKD group (HR 0.61; 95%CI 0.37–0.99; *p *< 0.05), but not in the DKD group. Patients on insulin in the NDKD group had a lower chance to be transplanted than patients without insulin (HR 0.61; 95%CI 0.44–0.85; *p *< 0.05), a relationship not found in the DKD group.

Finally, Table S5 shows the clinical heterogeneity of the NDKD group and suggests at least two subgroups with different outcomes. Patients in the NDKD group who accessed a transplant were younger and had far fewer comorbidities at inception than those who ultimately died.

**FIGURE 2:**
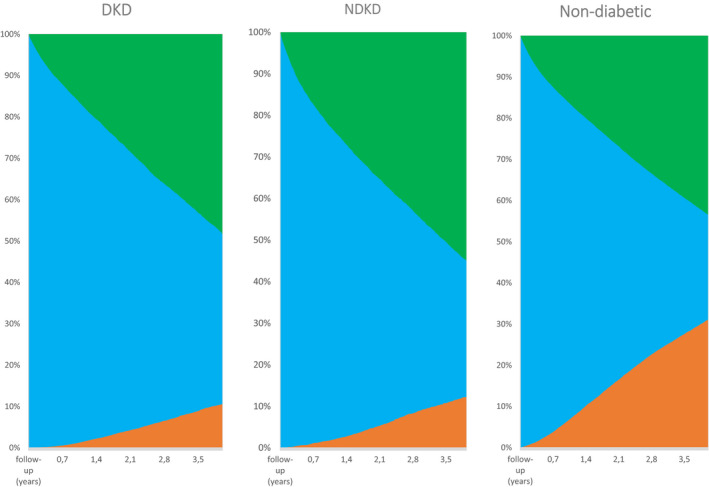
Cumulative incidence of transplantation and death, over four years [Colour figure can be viewed at wileyonlinelibrary.com]]

The hazard ratio of access to renal transplantation and the risk of death was studied in three competing risk models: without adjustment (M1), with adjustment for age (M2) and with adjustment for age, gender, MACE, cancer and chronic respiratory failure (M3) (Table 4). The risk of death was significantly higher in the NDKD group than in the DKD group, even after accounting for the competing risk of transplantation (M3) (NDKD/sdHR 1.22; 95%CI 1.18–1.27; *p* < 0.005 vs. DKD/sdHR 1.12; 95%CI 1.08–1.16; *p *< 0.005). Access to transplantation was significantly higher in the NDKD group than in the DKD group, even after accounting for the competing risk of death (Model 3) (NDKD/sdHR 0.55; 95%CI 0.50–0.60; *p *< 0.005 vs. DKD/sdHR 0.44; 95%CI 0.41–0.48; *p *< 0.005). Therefore, patients in the DKD group had a higher probability of remaining on dialysis (before death or transplantation) during the eight years after initiation of dialysis, as depicted in Figure 2.

**TABLE 4:** Comparison of the outcomes (death or renal transplantation) in the diabetic kidney disease (DKD) and non‐diabetic kidney disease (NDKD) groups versus the non‐diabetic group (reference group) with a competing risk model; calculation of sdHR (subdistribution hazard ratio) and csHR (cause‐specific hazard ratio)

	Model 1		Model 2		Model 3	
	sdHR	IC	sdHR	IC	sdHR	IC
Association with access to transplantation accounting for the competing risk of death						
Non‐diabetic (reference)	1.00		1.00		1.00	
DKD	0.29	0.27–0.30	0.44	0.42–0.47	0.44	0.41–0.48
NDKD	0.30	0.28–0.32	0.52	0.48–0.57	0.55	0.50–0.60
Association with the risk of death accounting for the competing risk of transplantation						
Non‐diabetic (reference)	1.00		1.00		1.00	
DKD	1.30	1.26–1.33	1.20	1.16–1.23	1.12	1.08–1.16
NDKD	1.58	1.53–1.63	1.31	1.27–1.35	1.22	1.18–1.27
	**csHR**	**IC**	**csHR**	**IC**	**csHR**	**IC**
Association with the risk of death accounting for the competing risk of transplantation						
Non‐diabetic (reference)	1.00		1.00		1.00	
DKD	1.13	1.10–1.16	1.12	1.09–1.15	1.06	1.03–1.10
NDKD	1.41	1.36–1.45	1.25	1.21–1.30	1.18	1.14–1.23

Abbreviations: DKD, diabetic kidney disease; M1, without adjustment; M2, with adjustment for age; M3, M2 + adjustment for gender, major adverse cardiovascular events, cancer, chronic respiratory failure; NDKD, non‐diabetic kidney disease.

## 4 DISCUSSION

In a cohort of more than 65,000 incident dialysis patients in the REIN registry, we examined the effect of the diagnosis of DKD vs. NDKD in diabetic patients on death and access to transplantation.

Type 2 diabetic patients were older and had more MACE and chronic respiratory failure than the non‐diabetic patients, which may be potentially related to a higher proportion of smoker and obese. The combination of smoking and diabetes multiplies cardiovascular risk and mortality and both the onset and the progression of diabetic nephropathy.^13^ The initiation of dialysis on a central venous catheter, in emergency or the ICU, was more common in diabetic patients, in contrast to guidelines promoting the timely placement of vascular access at eGFR 15 ml/min and even earlier in diabetic patients.^14^ The proportion of diabetic patients with a renal biopsy was dramatically low and significantly worse than in non‐diabetic patients. In our study, 62% of diabetic patients had ESKD attributed to diabetes which accounts for 25% of all patients starting renal replacement therapy. In a survey of 40 studies worldwide, the reported range was 17 to 83%, with an average of 64%.^15^

In our study, severe vision deficits were twice as common in the DKD group as in the NDKD group, suggesting that diabetic retinopathy was more common in the former group. We had no information on albuminuria before the initiation of dialysis. Still, there was no difference in serum albumin between DKD and NDKD, and both values were only marginally lower than in non‐diabetic patients. Diabetic nephropathy used to be responsible for nephrotic range albuminuria,^16^, ^17^ but recent studies reported lower rates of albuminuria,^18^ an observation partly explained by the broader prescription of blockers of the renin‐angiotensin system to delay progression.^19^ Insulin therapy was more frequently prescribed in the DKD group (75 vs. 50% in the NDKD group), suggesting either protracted uncontrolled diabetes or that insulin prescription may have biased the classification as DKD by the nephrologist. The proportion of patients receiving ESA was also higher in the DKD group, a relationship that suggested erythropoietin resistance and associated with increased cardiovascular mortality.^20^

In dialysis patients, diabetes was associated with increased mortality, especially from cardiovascular causes.^21^ Our study confirms these data with a 30–60% higher mortality risk among diabetic patients versus non‐diabetic patients, depending on the adjustment method. Among diabetic persons, those with NDKD had a higher risk of mortality than patients with DKD, which persisted after adjusting for confounders. However, when comparing the three groups, the survival rate dropped early and remained lower in the NDKD group. In contrast, the survival rate of DKD diverged from non‐diabetic patients only after the first year of follow‐up. The better survival overall in patients with DKD versus NDKD was unexpected because our initial hypothesis was that a diagnosis of diabetic nephropathy would reflect a more extensive arterial disease and higher mortality. Two older studies from the same group compared the risk of death in dialysis, according to DKD and NDKD diagnoses. In the Dutch NECOSAD study, 1,853 patients were followed for eight years, 15% in the DKD group and 6% in the NDKD group.^28^ Compared to the non‐diabetic group, mortality was higher in both DKD (HR 1.9) and NDKD (HR 2.1) groups but similar between the diabetic groups (HR 1.06). In the European EDTA database (excluding France) in 15,419 patients and a 5‐year follow‐up,^10^ the mortality rate was higher in the DKD compared to the NDKD group after adjustment for age, sex, country, cancer, cardiovascular comorbidities (HR 1.20; 95%CI 1.10–1.30). The NECOSAD study was probably underpowered to show any difference, but even in the second study, the proportion of diabetic patients was much lower than in our cohort. In our study, the proportion of DKD and NDKD patients was similar, but diabetic patients overall had a 30% lower mortality than in the EDTA cohort. These discrepancies may be related to outdated standard of care for diabetes or higher mortality rate in the background population as the EDTA cohort mainly originated from northern Europe.

Access to kidney transplantation was more than twice as low in type 2 diabetic patients compared to dialysis patients without diabetes, in agreement with the literature.^21^, ^22^, ^23^ In DKD patients, cardiovascular comorbidities would be expected to hinder access to transplantation. Accordingly, patients with disabilities such as peripheral arterial disease complicated by amputation and motor deficits secondary to stroke had reduced access to transplantation. In the NECOSAD study, 33% of non‐diabetic patients received a renal transplant vs. 17% in the DKD group and 8% in the NDKD group. Accessibility to transplant was overall better in their cohort than in ours. The proportion of transplant recipients was higher in the DKD group than in the NDKD group, in contrast to our cohort.^9^ In the EDTA study, transplantation was 36% in the non‐diabetic group, vs. 13% in the DKD or the NDKD group.^10^ In our study, the determinants of access to transplantation in the NDKD and DKD groups were partly similar. However, a history of non‐kidney transplants was strongly positively associated with kidney transplantation in the NDKD group, unlike the DKD group. In contrast, NDKD patients with congestive heart failure or those treated with insulin had a reduced chance of transplantation. The negative effect of the insulin treatment raises some questions about this association, which may include fear for increased risk of hypoglycaemia, the anticipation of diabetes more difficult to manage with glucocorticoids or the perception that a diabetic patient on insulin has more extensive vascular damage.

In our study, patients with NDKD had both a higher risk of death and more access to kidney transplantation paradoxically. Type 2 diabetic patients have better survival after kidney transplant than diabetic patients staying on dialysis,^24^, ^25^ resulting from selection bias.^25^ Also, transplantation is a competitive risk for death in dialysis patients, a bias not adequately addressed in the Cox model.^26^ Using a competing risk model^27^, ^28^ accounting for kidney transplantation, the risk of death was still higher in NDKD patients than in the DKD group. However, after accounting for the competing risk of death, access to transplantation was also higher in patients with NDKD. We hypothesized that the NDKD group is heterogeneous, including a subgroup of sicker patients with early mortality and another subset in better shape with faster access to transplantation than DKD patients. Accordingly, transplanted patients were 16 years younger and had fewer comorbidities, particularly congestive heart failure and peripheral artery disease, than the patients prone to death, and may represent a group of patients with primary renal diseases and superimposed diabetes.

Some limitations of our study should bear in mind. The data were collected at the inception of dialysis; hence, we had no information on the predialysis course (diabetes duration, metabolic control, hypertension, eGFR decline rate, albuminuria). Also, the distinction between DKD and NDKD relied on nephrologist discretion and was generally based on clinical evidence only, as indicated by the low rate of renal biopsy. The final diagnosis retained in the register could have been influenced by local coding practices or the background prevalence of diabetes depending on the region.

Our study has some strengths, however, relying on an exhaustive nationwide registry and a long‐term follow‐up. All items at the inception of dialysis were prespecified, and outcomes were collected prospectively. Finally, our analyses used extensive adjustments and accounted for the competitive risk between death and renal transplantation.

## 5 CONCLUSIONS

In diabetic patients starting dialysis, DKD vs. NDKD was associated with distinct clinical patterns and outcomes. Patients with DKD had more vascular complications and disabilities and reduced access to kidney transplantation. NDKD‐rated patients had more cancer, liver diseases, arrhythmias and a higher risk of mortality than DKD‐rated patients. However, a subgroup of NDKD patients has better access to kidney transplantation, which was associated with an identifiable favourable clinical pattern. This distinction should be accounted for in the plan of care of diabetic patients starting dialysis.

## CONFLICT OF INTEREST

The authors declared no conflict of interest in this work.

## AUTHORS’ CONTRIBUTIONS

AD was involved in the writing and statistical analysis. TH and TK contributed to the conceptualization of the study, writing and proofreading. CC contributed to the statistical analyses. JG, DC, FC, JM and NK were involved in the acquisition of the data, and proofreading.

## Supporting information

Supplementary Material

## Data Availability

This study was conducted using data from the REIN registry (Nephrology Information and Epidemiological Network). These data can be made available by request to the Scientific Council of the REIN registry.

## References

[1] Ogurtsova K , da Rocha Fernandes JD , Huang Y , et al. IDF Diabetes Atlas: global estimates for the prevalence of diabetes for 2015 and 2040. Diabetes Res Clin Pract. 2017;128:40‐50. 28437734 10.1016/j.diabres.2017.03.024

[2] Jha V , Garcia‐garcia G , Iseki K , et al. Global kidney disease 3 chronic kidney disease: global dimension and perspectives. Lancet. 2013;382:260‐272. 23727169 10.1016/S0140-6736(13)60687-X

[3] Tuttle KR , Bakris GL , Bilous RW , et al. Diabetic kidney disease: a report from an ADA consensus conference. Diabetes Care. 2014;37:2864‐2883. 25249672 10.2337/dc14-1296 PMC4170131

[4] Afkarian M , Zelnick LR , Hall YN , et al. Clinical manifestations of kidney disease among US adults with diabetes, 1988–2014. J Am Med Assoc. 2016;316:602‐610. 10.1001/jama.2016.10924 PMC5444809 27532915

[5] Zelnick LR , Weiss NS , Kestenbaum BR , et al. Diabetes and CKD in the United States population. Clin J Am Soc Nephrol. 2017;12:2009‐2014. 10.2215/CJN.03700417 PMC5718269 29054846

[6] Assogba FGA , Couchoud C , Hannedouche T , et al. Trends in the epidemiology and care of diabetes mellitus‐related end‐stage renal disease in France, 2007–2011. Diabetologia. 2014;57:718‐728. 24496924 10.1007/s00125-014-3160-9

[7] Lassalle M , Monnet E , Ayav C , Hogan J , Moranne O , Couchoud C . 2017 Annual report digest of the renal epidemiology information network (REIN) registry. Transpl Int. 2019;32:892‐902. 31148236 10.1111/tri.13466

[8] Sørensen VR , Mathiesen ER , Heaf J , Feldt‐Rasmussen B . Improved survival rate in patients with diabetes and end‐stage renal disease in Denmark. Diabetologia. 2007;50:922‐929. 17333109 10.1007/s00125-007-0612-5

[9] Schroijen MA , Dekkers OM , Grootendorst DC , et al. Survival in dialysis patients is not different between patients with diabetes as primary renal disease and patients with diabetes as a co‐morbid condition. BMC Nephrol. 2011;12:69. 22182634 10.1186/1471-2369-12-69 PMC3259092

[10] Schroijen MA , Van De Luijtgaarden MWM , Noordzij M , et al. Survival in dialysis patients is different between patients with diabetes as primary renal disease and patients with diabetes as a co‐morbid condition. Diabetologia. 2013;56:1949‐1957. 23771173 10.1007/s00125-013-2966-1

[11] Couchoud C , Stengel B , Landais P , et al. The renal epidemiology and information network (REIN): a new registry for end‐stage renal disease in France. Nephrol Dial Transplant. 2006;21:411‐418. 16234286 10.1093/ndt/gfi198

[12] Fine JP , Gray RJ . A Proportional hazards model for the subdistribution of a competing risk. J Am Stat Assoc. 1999;94:496‐509.

[13] Iwai T , Miyazaki M , Yamada G , et al. Diabetes mellitus as a cause or comorbidity of chronic kidney disease and its outcomes: the Gonryo study. Clin Exp Nephrol. 2018;22:328‐336. 28752289 10.1007/s10157-017-1451-4

[14] Gallieni M , Hollenbeck M , Inston N , et al. Clinical practice guideline on peri‐ and postoperative care of arteriovenous fistulas and grafts for haemodialysis in adults. Nephrol Dial Transplant. 2012;34:II1‐II42. 10.1093/ndt/gfz072 31192372

[15] Anders H‐J , Huber TB , Isermann B , Schiffer M . CKD in diabetes: diabetic kidney disease versus non‐diabetic kidney disease. Nat Rev Nephrol. 2018;14:s361‐377. 10.1038/s41581-018-0001-y 29654297

[16] Mogensen CE . Introduction: diabetes mellitus and the kidney. Kidney Int. 1982;21:673‐675. 7109457 10.1038/ki.1982.79

[17] Alicic R , Rooney M , Tuttle K . Diabetic kidney disease: challenges, progress, and possibilities. Clin J Am Soc Nephrol. 2017;12:2032‐2045. 28522654 10.2215/CJN.11491116 PMC5718284

[18] Porrini E , Ruggenenti P , Mogensen CE , et al. Non‐proteinuric pathways in loss of renal function in patients with type 2 diabetes. Lancet Diabetes Endocrinol. 2015;3:382‐391. 25943757 10.1016/S2213-8587(15)00094-7

[19] Molitch ME , Adler AI , Flyvbjerg A , et al. Diabetic kidney disease‐A clinical update from kidney disease: improving global outcomes (KDIGO). Kidney Int. 2015;87:20‐30. 24786708 10.1038/ki.2014.128 PMC4214898

[20] Solomon SD , Uno H , Lewis EF , et al. Erythropoietic response and outcomes in kidney disease and type 2 diabetes. N Engl J Med. 2010;363:1146‐1155. 20843249 10.1056/NEJMoa1005109

[21] Burmeister JE , Mosmann CB , Costa VB , et al. Prevalence of cardiovascular risk factors in hemodialysis patients–The CORDIAL study. Arq Bras Cardiol. 2014;102(5):473‐480. 24759948 10.5935/abc.20140048 PMC4051450

[22] Van Dijk PCW , Jager KJ , Stengel B , Gronhagen‐Riska C , Feest TG , Douglas BJ . Renal replacement therapy for diabetic end‐stage renal disease: data from 10 registries in Europe (1991–2000). Kidney Int. 2005;67:1489‐1499. 15780102 10.1111/j.1523-1755.2005.00227.x

[23] Bayat S , Frimat L , Thilly N , Loos C , Briançon S , Kessler M . Medical and non‐medical determinants of access to renal transplant waiting list in a French community‐based network of care. Nephrol Dial Transplant. 2006;21:2900‐2907. 16861245 10.1093/ndt/gfl329

[24] Patibandla BK , Narra A , DeSilva R , Chawla V , Goldfarb‐Rumyantzev AS . Access to renal transplantation in the diabetic population‐effect of comorbidities and body mass index. Clin Transplant. 2012;26:E307‐E315. 22686955 10.1111/j.1399-0012.2012.01661.x PMC3756087

[25] Wolfe RA , Ashby VB , Milford EL , et al. Comparison of mortality in all patients on dialysis, patients on dialysis awaiting transplantation, and recipients of a first cadaveric transplant. N Engl J Med. 1999;341:1725‐1730. 10580071 10.1056/NEJM199912023412303

[26] Pérez‐Sáez MJ , Pascual J , Navarro‐González JF , Luis D . Kidney transplantation in the diabetic patient. J Clin Med. 2015;4:1269‐1280. 26239558 10.3390/jcm4061269 PMC4484999

[27] Lim HJ , Zhang X , Dyck R , Osgood N . Methods of competing risks analysis of end‐stage renal disease and mortality among people with diabetes. BMC Med Res Methodol. 2010;10:97. 20964855 10.1186/1471-2288-10-97 PMC2988010

[28] Warnock DG . Competing risks: you only die once. Nephrol Dial Transplant. 2016;31:1033‐1035. 26908777 10.1093/ndt/gfv455 PMC6292449

