# Supplementary material for: Diabetic kidney disease versus non‐diabetic kidney disease in type 2 diabetic patients on dialysis: An observational cohort
Source: Endocrinol Diabetes Metab. 2022 Apr 30;5(4):e00281. doi: 10.1002/edm2.281 (PMC9258992; doi:10.1002/edm2.281)
Supplement: Supplementary file 1 — Supplementary Material [file EDM2-5-e00281-s001.docx]

**Diabetic kidney disease versus non-diabetic kidney disease in type 2 diabetic patients on dialysis: An observationnal cohort (by A. Delautre & al.)**

**Supplemental Figures and Tables**

**Supplemental Figure 1 : STROBE flowchart diagram**

**Supplemental Table 1 : Hazard ratio of mortality in diabetic patients (all, DKD, NDKD) versus non-diabetic patients over the entire period without censoring at transplantation. Multivariate analysis with a Cox model.**

**Supplemental Table 2: Causes of death according to the coding of diabetic patients in DKD or NDKD**

**Supplemental Table 3: Clinical determinants associated with the probability of transplantation in diabetic patients starting dialysis**

**Supplemental Table 4: Clinical determinants associated with the probability of transplantation: DKD vs NDKD.**

**Supplemental Table 5: Clinical characteristics associated with transplantation or death in NDKD patients.**

**Supplemental Figure 1: STROBE flowchart diagram
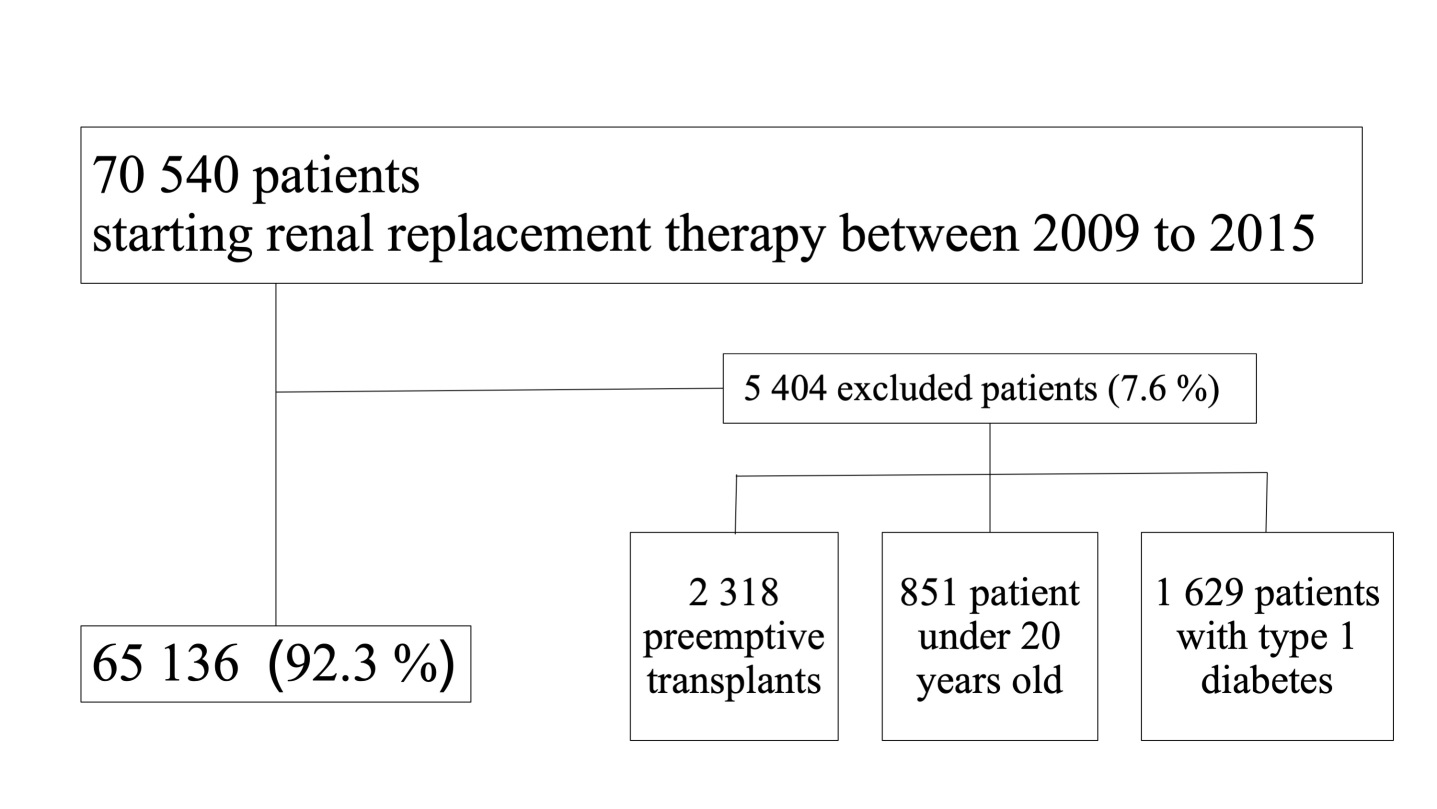
**

**Supplemental Table 1: Hazard ratio of all-cause mortality in diabetic patients (all, DKD, NDKD) versus non-diabetic patients over the entire period without censoring at transplantation. Multivariate analysis with a Cox model.**

|  | *Model 1* | | | | *Model 2* | | | | *Model 3* | | | |
| --- | --- | --- | --- | --- | --- | --- | --- | --- | --- | --- | --- | --- |
|  | HR | IC | | | HR | IC | | | HR | IC | | |
| Non-diabetic patients | 1.00 |  |  |  | 1.00 |  |  |  | 1.00 |  |  |  |
| All Diabetic patients | 1.39 | 1.36 | - | 1.43 | 1.23 | 1.20 | - | 1.26 | 1.15 | 1.12 | - | 1.19 |
| DKD | 1.29 | 1.26 | - | 1.33 | 1.18 | 1.15 | - | 1.22 | 1.11 | 1.07 | - | 1.15 |
| NDKD | 1.58 | 1.53 | - | 1.63 | 1.30 | 1.26 | - | 1.34 | 1.21 | 1.17 | - | 1.26 |

*Legend*: *DKD: Diabetic kidney disease, NDKD : Non-diabetic kidney disease*

*M1: without adjustment*

*M2: with adjustment for age*

*M3: M2 + adjustment for gender, major adverse cardiovascular events, cancer, chronic respiratory failure*

*HR: hazard ratio*

*CI: 95% confidence interval*

**Supplemental Table 2: Causes of death according to the coding of diabetic patients as DKD or NDKD**

| Causes of death | DKD | NDKD |
| --- | --- | --- |
| Unknown cause (N & %) | 2 519 | 1 587 |
|  | 33% | 30% |
| Infectious diseases (N & %) | 1 096 | 663 |
|  | 14% | 13% |
| Other known cause (N & %) | 914 | 654 |
|  | 12% | 13% |
| Other diseases of the circulatory system  (N & %) | 619 | 377 |
|  | 8% | 7% |
| Cancer (N & %) | 455 | 491 |
|  | 6% | 9% |
| Cachexia (N & %) | 521 | 405 |
|  | 7% | 8% |
| Heart diseases (N & %) | 991 | 746 |
|  | 13% | 14% |
| Cerebrovascular disease (N & %) | 325 | 199 |
|  | 4.3% | 3.8% |
| Liver disease (N & %) | 39 | 50 |
|  | 0.5% | 1.0% |
| Kidney disease & hyperkalemia (N & %) | 89 | 55 |
|  | 1.2% | 1.1% |
| Total | 7 568 | 5 227 |

*Legends*: *DKD: Diabetic kidney disease, NDKD: Non-diabetic kidney disease*

**Supplemental Table 3: Clinical determinants associated with the probability of all-cause mortality in diabetic patients starting dialysis. Multivariate analysis with the Cox model and with a competing risk model; Calculation of sdHR (Sub-distribution Hazard ratio) and csHR (cause-specific Hazard ratio)**

| Characteristics | **HR** | CI | p | **csHR** | CI | p | **sdHR** | CI | p |
| --- | --- | --- | --- | --- | --- | --- | --- | --- | --- |
|  |  |  |  |  |  |  |  |  |  |
| Age (per year) | 1.04 | 1.03-1.04 | < 0.001 | 1.04 | 1.03-1.04 | < 0.001 | 1.04 | 1.04-1.05 | < 0.001 |
| Female gender | 0.92 | 0.80-1.06 | 0.251 | 0.92 | 0.80-1.06 | 0.262 | 0.93 | 0.80-1.07 | 0.289 |
| BMI (Body Mass Index) (per 1 unit) | 1.02 | 1.01-1.03 | < 0.001 | 1.02 | 1.01-1.03 | < 0.001 | 1.02 | 1.02-1.03 | < 0.001 |
| Serum Albumin (per g/L) | 0.98 | 0.97-0.99 | < 0.001 | 0.98 | 0.97-0.99 | < 0.001 | 0.98 | 0.97-0.99 | < 0.001 |
| Hemoglobin (per g/dL) | 0.99 | 0.96-1.02 | 0.430 | 0.99 | 0.96-1.02 | 0.408 | 0.99 | 0.96-1.02 | 0.420 |
| eGFR (per ml/min/1.73m2) | 1.01 | 1.00-1.01 | 0.011 | 1.01 | 1.00-1.01 | 0.024 | 1.01 | 1.00-1.01 | 0.027 |
| Renal biopsy | 0.89 | 0.76-1.03 | 0.107 | 0.89 | 0.77-1.03 | 0.124 | 0 .89 | 0.76-1.04 | 0.136 |
| **MACE** | 1.08 | 0.93-1.26 | 0.299 | 1.07 | 0.92-1.25 | 0.397 | 1.09 | 0.93-1.27 | 0.283 |
| Ischemic heart disease | 1.08 | 0.98-1.20 | 0.137 | 1.09 | 0.98-1.20 | 0.117 | 1.09 | 0.98-1.21 | 0.111 |
| Congestive Heart Failure | 1.27 | 1.15-1.41 | < 0.001 | 1.28 | 1.15-1.42 | < 0.001 | 1.28 | 1.15-1.42 | < 0.001 |
| Arrhythmia | 1.34 | 1.21-1.48 | < 0.001 | 1.32 | 1.19-1.47 | < 0.001 | 1.34 | 1.20-1.49 | < 0.001 |
| Abdominal aortic aneurysm | 1.07 | 0.85-1.35 | 0.566 | 1.07 | 0.85-1.35 | 0.543 | 1.08 | 0.86-1.34 | 0.522 |
| Peripheral arterial disease | 1.27 | 1.15-1.41 | < 0.001 | 1.27 | 1.14-1.41 | < 0.001 | 1.30 | 1.17-1.45 | <0.001 |
| Stroke | 1.02 | 0.90-1.16 | 0.769 | 1.02 | 0.90-1.16 | 0.756 | 1.03 | 0.90-1.18 | 0.626 |
| **Disability** |  |  |  |  |  |  |  |  |  |
| Disability | 1.31 | 1.11-1.55 | 0.002 | 1.30 | 1.10-1.54 | 0.003 | 1.32 | 1.09-1.60 | 0.005 |
| Amputation | 1.22 | 0.95-1.58 | 0.124 | 1.20 | 0.93-1.55 | 0.164 | 1.23 | 0.92-1.66 | 0.166 |
| Hemiplegia or paraplegia | 0.88 | 0.58-1.27 | 0.445 | 0.88 | 0.59-1.32 | 0.542 | 0.88 | 0.55-1.42 | 0.610 |
| Severe vision impairment | 0.76 | 0.61-0.96 | 0.022 | 0.76 | 0.60-0.96 | 0.022 | 0.75 | 0.59-0.96 | 0.021 |
| Severe behavioral disorders | 0.99 | 0.75-0.32 | 0.963 | 0.97 | 0.73-1.29 | 0.854 | 1.00 | 0.73-1.38 | 0.983 |
| **Other comorbidity** |  |  |  |  |  |  |  |  |  |
| Smoking | 1.13 | 1.02-1.26 | 0.023 | 1.11 | 0.99-1.24 | 0.066 | 1.12 | 0.99-1.25 | 0.054 |
| Chronic respiratory Disease | 1.27 | 1.14-1.43 | < 0.001 | 1.26 | 1.12-1.42 | < 0.001 | 1.25 | 1.11-1.41 | < 0.001 |
| Cancer | 1.57 | 1.36-1.80 | < 0.001 | 1.53 | 1.33-1.76 | < 0.001 | 1.58 | 1.35-1.85 | < 0.001 |
| Liver disease | 1.60 | 1.29-1.98 | < 0.001 | 1.59 | 1.28-1.98 | < 0.001 | 1.64 | 1.31-2.04 | < 0.001 |
| Other transplantation than kidney | 1.35 | 0.93-1.95 | 0.113 | 1.63 | 1.12-1.36 | 0.010 | 1.50 | 1.05-2.14 | 0.027 |
| HIV infection or AIDS | 0.38 | 0.09-1.52 | 0.173 | 0.41 | 0.10-1.63 | 0.203 | 0.34 | 0.79-1.46 | 0.147 |
| **Treatment** |  |  |  |  |  |  |  |  |  |
| First dialysis in emergency | 1.02 | 0.91-1.15 | 0.695 | 1.04 | 0.92-1.17 | 0.510 | 1.05 | 0.92-1.19 | 0.492 |
| First HD on catheter | 1.07 | 0.96-1.19 | 0.227 | 1.07 | 0.96-1.19 | 0.232 | 1.08 | 0.97-1.21 | 0.160 |
| First HD in intensive care unit | 0.99 | 0.82-1.19 | 0.924 | 0.97 | 0.81-1.18 | 0.787 | 0.98 | 0.81-1.21 | 0.873 |
| First KRT modality: HD vs PD | 1.15 | 0.63-2.12 | 0.643 | 1.27 | 0.67-2.39 | 0.467 | 1.28 | 0.67-2.43 | 0.452 |
| ESA treatment | 1.04 | 0.95-1.14 | 0.407 | 1.05 | 0.95-1.15 | 0.349 | 1.04 | 0.95-1.15 | 0.391 |
| Insulin treatment | 1.08 | 0.97-1.19 | 1.161 | 1.06 | 0.96-1.18 | 0.258 | 1.08 | 0.97-1.20 | 0.172 |
| Time between fistula and HD (month) | 1.00 | 1.00-1.00 | 0.827 | 1.00 | 0.99-1.00 | 0.568 | 1.00 | 0.99-1.00 | 0.721 |

*Legends: HD: Hemodialysis; PD: peritoneal dialysis;**KRT: Kidney replacement therapy, DKD: Diabetic kidney disease; NDKD: Non diabetic kidney disease;, MACE: major adverse cardiovascular events; Smoking: smoker and ex-smoker; HIV: human immunodeficiency virus, AIDS : Acquired Immunodeficiency Syndrome ; ESA: Erythropoietin Stimulating Agent*

**Supplemental Table 4: Clinical determinants associated with the probability of transplantation in diabetic patients starting dialysis. Multivariate analysis with the Cox model and with a competing risk model; Calculation of sdHR (Sub-distribution Hazard ratio) and csHR (cause-specific)**

| Characteristics | **csHR** | CI | p | **sdHR** | CI | p |
| --- | --- | --- | --- | --- | --- | --- |
|  |  |  |  |  |  |  |
| Age (per year) | **0.94** | **0.93-0.94** | **< 0.001** | **0.93** | **0.92-0.94** | **< 0.001** |
| Female gender | 0.98 | 0.73-1.31 | 0.882 | 0.96 | 0.71-1.29 | 0.770 |
| BMI (Body Mass Index) (per 1 unit) | 0.93 | 0.81-1.06 | 0.280 | 0.94 | 0.83-1.06 | 0.314 |
| Serum Albumin (per g/L) | **1.03** | **1.01-1.05** | **0.001** | **1.03** | **0.01-1.05** | **< 0.001** |
| Hemoglobin (per g/dL) | 1.03 | 0.96-1.10 | 0.391 | 1.05 | 0.98-1.13 | 0.139 |
| eGFR (per ml/min/1.73m2) | **0.96** | **0.93-0.99** | **0.008** | **0.95** | **0.91-0.99** | **0.036** |
| Renal biopsy | 0.83 | 0.64-1.09 | 0.183 | 0.87 | 0.66-1.14 | 0.315 |
| **MACE** | 1.35 | 0.93-1.96 | 0.117 | 1.36 | 0.94-1.97 | 0.101 |
| Ischemic heart disease | 0.80 | 0.57-1.10 | 0.167 | 0.76 | 0.56-1.05 | 0.096 |
| Congestive Heart Failure | **0.69** | **0.49-0.97** | **0.033** | **0.67** | **0.48-0.94** | **0.019** |
| Arrhythmia | **0.48** | **0.32-0.71** | **< 0.001** | **0.44** | **0.30-0.64** | **< 0.001** |
| Abdominal aortic aneurysm | 0.65 | 0.24-1.78 | 0.408 | 0.64 | 0.24-1.73 | 0.382 |
| Peripheral arterial disease | **0.37** | **0.26-0.54** | **< 0.001** | **0.36** | **0.25-0.52** | **< 0.001** |
| Stroke | **0.57** | **0.37-0.88** | **0.012** | **0.57** | **0.36-0.89** | **0.013** |
| **Disabilities** |  |  |  |  |  |  |
| Disability | 0.64 | 0.37-1.12 | 0.120 | 0.62 | 0.35-1.09 | 0.095 |
| Amputation | 0.40 | 0.09-1.74 | 0.223 | 0.38 | 0.09-1.61 | 0.192 |
| Hemiplegia or paraplegia | 1.58 | 0.45-5.62 | 0.477 | 1.42 | 0.40-5.05 | 0.585 |
| Severe vision impairment | 1.55 | 0.78-3.07 | 0.214 | 1.53 | 0.76-3.10 | 0.231 |
| Severe behavioral disorders | 0.47 | 0.16-1.43 | 0.185 | 0.51 | 1.18-1.41 | 0.216 |
| **Other comorbidity** |  |  |  |  |  |  |
| Smoking | 1.09 | 0.87-1.36 | 0.469 | 1.10 | 0.88-1.38 | 0.408 |
| Chronic respiratory Disease | 1.10 | 0.80-1.52 | 0.540 | 0.99 | 0.70-1.38 | 0.936 |
| Cancer | **0.29** | **0.15-0.56** | **< 0.001** | **0.25** | **0.11-0.54** | **< 0.001** |
| Liver disease | 1.10 | 0.67-1.81 | 0.713 | 1.00 | 0.61-1.64 | 0.994 |
| Other transplantation than kidney | **2.06** | **1.13-3.79** | **0.019** | 1.36 | 0.62-3.00 | 0.445 |
| HIV infection or AIDS | 1.37 | 0.50-3.74 | 0.540 | 1.45 | 0.64-3.26 | 0.374 |
| **Treatment** |  |  |  |  |  |  |
| First dialysis in emergency | 0.82 | 0.60-1.12 | 0.218 | 0.84 | 0.61-1.15 | 0.283 |
| First HD on catheter | **0.65** | **0.49-0.86** | **0.002** | **0.65** | **0.49-0.86** | **O.002** |
| First HD in ICU | 1.20 | 0.72-2.02 | 0.283 | 1.17 | 0.69-1.97 | 0.555 |
| First KRT modality : HD Vs PD | 1.74 | 0.54-5.67 | 0.355 | 1.75 | 0.65-4.69 | 0.267 |
| ESA treatment | 1.05 | 0.84-1.30 | 0.677 | 1.04 | 0.83-1.30 | 0.745 |
| Insulin treatment | **0.75** | **0.60-0.94** | **0.12** | **0.75** | **0.60-0.95** | **0.015** |
| Time between fistula and HD (month) | 1.00 | 0.99-1.00 | 0.681 | 1.00 | 0.99-1.00 | 0.834 |

*Legends: MACE: major adverse cardiovascular events; Smoking: smoker and ex-smoker;* *AIDS: Acquired Immunodeficiency Syndrome; HD & PD: Hemodialysis & peritoneal dialysis; KRT: Kidney replacement therapy ; ESA: Erythropoietin Stimulating Agent*

**Supplemental Table 5: Clinical determinants associated with the probability of transplantation: DKD vs NDKD. Multivariate analysis with the Cox model.**

|  | DKD | | | | NDKD | | | |
| --- | --- | --- | --- | --- | --- | --- | --- | --- |
|  | HR | IC | | | HR | IC | | |
| Serum Albumin (per g/L) | 1.02 | 1.00 | - | 1.04 | 1.04 | 1.02 | - | 1.06 |
| First HD on central catheter | 0.69 | 0.53 | - | 0.92 | 0.57 | 0.39 | - | 0.83 |
| BMI (per unit) | 0.95 | 0.94 | - | 0.97 | 0.96 | 0.94 | - | 0.98 |
| Congestive Heart failure | NS | | | | 0.61 | 0.37 | - | 0.99 |
| Age (per year) | 0.93 | 0.92 | - | 0.94 | 0.93 | 0.92 | - | 0.94 |
| Peripheral Arterial Disease | 0.60 | 0.43 | - | 0.84 | 0.36 | 0.21 | - | 0.62 |
| Cancer | 0.39 | 0.19 | - | 0.80 | 0.24 | 0.11 | - | 0.54 |
| Disabilities | 0.60 | 0.43 | - | 0.83 | 0.49 | 0.29 | - | 0.84 |
| Other transplants | NS | | | | 2.40 | 1.32 | - | 4.34 |
| Female gender | 0.70 | 0.54 | - | 0.88 | NS | | | |
| Insulin treatment | NS | | | | 0.72 | 0.54 | - | 0.95 |

*Legends*: *DKD: Diabetic kidney disease, NDKD: Non-diabetic kidney disease*

**Supplemental Table 6: Clinical characteristics associated with transplantation or death in NDKD patients*.**

| **Clinical characteristics** at initiation of dialysis | **Transplanted NDKD**  n=717 (7.1 %) | **Deceased NDKD**  n = 5 207  (51.4 %) | p* |
| --- | --- | --- | --- |
|  |  |  |  |
| Age, mean | 59.8 | 75.8 | < 0.005 |
| Male,% | 69% | 67% | NS |
| BMI, mean | 27.8 | 27.6 | NS |
| Serum Albumin, median | 34.9 | 31.8 | < 0.005 |
| Hemoglobin, median | 10.3 | 10.1 | NS |
| eGFR, mean | 9.1 | 12.2 | < 0.005 |
| Renal biopsy,% | 32 | 12 | < 0.005 |
| **Major adverse cardiovascular events (MACE)** | 40% | 80% | < 0.005 |
| MACE, average | 0.6 | 1.8 | < 0.005 |
| Ischemic heart disease,% | 20% | 40% | < 0.005 |
| Congestive Heart failure, % | 14% | 44% | < 0.005 |
| Arrhythmia,% | 9% | 38% | < 0.005 |
| Abdominal aortic aneurysm,% | 3% | 6% | < 0.005 |
| Peripheral arterial disease,% | 10% | 43% | < 0.005 |
| Stroke,% | 6% | 16% | < 0.005 |
| **Disabilities** |  |  |  |
| Disability,% | 5% | 19% | < 0.005 |
| Amputation,% | 1% | 3% | < 0.005 |
| Hemiplegia or paraplegia,% | 0.5% | 2% | < 0.005 |
| Severe vision loss,% | 1% | 3% | < 0.005 |
| Behavior disorder, % | 1% | 5% | < 0.005 |
| **Other comorbidities** |  |  |  |
| Smoking,% | 44% | 47% | NS |
| Chronic respiratory failure,% | 9% | 22% | < 0.005 |
| Cancer,% | 2% | 15% | < 0.005 |
| Hepatopathy,% | 6% | 7% | < 0.005 |
| Other transplants than kidneys,% | 6.5% | 2% | < 0.005 |
| HIV infection or AIDS,% | 1.5% | 0.5% | < 0.005 |
| First dialysis in emergency,% | 22% | 36% | < 0.005 |
| First dialysis on central catheter,% | 43% | 64% | < 0.005 |
| First dialysis in intensive care unit,% | 6% | 12% | < 0.005 |
| Hemodialysis vs Peritoneal Dialysis,% | 89% | 89% | NS |
| ESA before dialysis,% | 49% | 47% | NS |
| Insulin,% | 43% | 58% | < 0.005 |
| Delay of fistula creation before dialysis, average (mo) | 5.8 | 5.6 | NS |

*Legends*: *MACE: major adverse cardiovascular events, Smoking (current smoker and ex-smoker), HIV: human immunodeficiency virus, AIDS : Acquired Immunodeficiency Syndrome, HD & PD: Hemodialysis & peritoneal dialysis DKD: Diabetic kidney disease, NDKD: Non-diabetic kidney disease ; ESA: Erythropoietin Stimulating Agent.*

* We adjusted on the following criteria: first emergency dialysis session, first session on catheter, chronic respiratory insufficiency, MACE, heart failure, coronary artery disease, peripheral arterial disease, cancer, hepatopathy, disability, gender, age, serum albumin and hemoglobin at the initiation of dialysis, BMI, kidney biopsy, insulin treatment, modality of KRT, delay between fistula creation and the onset of hemodialysis, other transplantation than the kidney. Variables retained in the multivariate analysis were those with a P <0.20 in the univariate analysis. Hemoglobin concentration was "forced" into model despite a P > 0.20 as it is an accepted prognosis factor in the dialysis population.
